# Drug-loaded adhesive microparticles for biofilm prevention on oral surfaces†

**DOI:** 10.1039/d4tb00134f

**Published:** 2024-04-29

**Authors:** Min Jun Oh, Jae-Hyun Kim, Jaekyoung Kim, Sunghee Lee, Zhenting Xiang, Yuan Liu, Hyun Koo, Daeyeon Lee

**Affiliations:** a Department of Chemical and Biomolecular Engineering, University of Pennsylvania Philadelphia Pennsylvania 19104 USA daeyeon@seas.upenn.edu; b Department of Orthodontics, School of Dental Medicine, University of Pennsylvania Philadelphia Pennsylvania 19104 USA koohy@upenn.edu; c Center for Innovation & Precision Dentistry, School of Dental Medicine and School of Engineering and Applied Sciences, University of Pennsylvania Philadelphia Pennsylvania 19104 USA

## Abstract

The oral cavity, a warm and moist environment, is prone to the proliferation of microorganisms like *Candida albicans* (*C. albicans*), which forms robust biofilms on biotic and abiotic surfaces, leading to challenging infections. These biofilms are resistant to conventional treatments due to their resilience against antimicrobials and immune responses. The dynamic nature of the oral cavity, including the salivary flow and varying surface properties, complicates the delivery of therapeutic agents. To address these challenges, we introduce dendritic microparticles engineered for enhanced adhesion to dental surfaces and effective delivery of antifungal agents and antibiofilm enzymes. These microparticles are fabricated using a water-in-oil-in-water emulsion process involving a blend of poly(lactic-*co*-glycolic acid) (PLGA) random copolymer (RCP) and PLGA-*b*-poly(ethylene glycol) (PLGA-*b*-PEG) block copolymer (BCP), resulting in particles with surface dendrites that exhibit strong adhesion to oral surfaces. Our study demonstrates the potential of these adhesive microparticles for oral applications. The adhesion tests on various oral surfaces, including dental resin, hydroxyapatite, tooth enamel, and mucosal tissues, reveal superior adhesion of these microparticles compared to conventional spherical ones. Furthermore, the release kinetics of nystatin from these microparticles show a sustained release pattern that can kill *C. albicans*. The biodegradation of these microparticles on tooth surfaces and their efficacy in preventing fungal biofilms have also been demonstrated. Our findings highlight the effectiveness of adhesive microparticles in delivering therapeutic agents within the oral cavity, offering a promising approach to combat biofilm-associated infections.

## Abstract 2

This study introduces dendritic microparticles from PLGA and PLGA-*b*-PEG for oral antifungal delivery. They adhere to oral surfaces, offer sustained release, and prevent biofilms, while being biodegradable, promising for treating oral infections.

## Graphical abstract

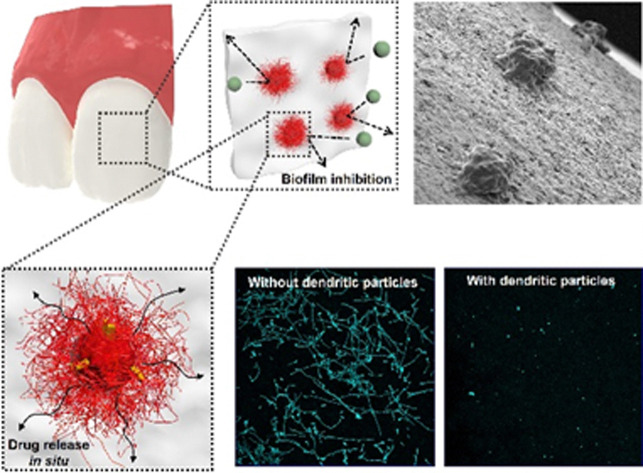

## Introduction

The oral cavity provides a hospitable, warm and moist environment for the proliferation of undesirable microorganism growth.^1^ For example, *Candida albicans* (*C. albicans*), an opportunistic fungal pathogen, can overgrow in the mouth and form robust biofilms—structured communities of microorganisms—adhering to tooth and mucosal surfaces, causing costly oral infections.^2^ These biofilms exhibit extraordinary resistance to antimicrobials and host immune responses, rendering conventional treatment approaches less effective and posing significant challenges to eradication.^3^ Furthermore, the transition of *C. albicans* from yeast to hyphal forms augments its virulence and resistance to antifungal agents, promoting its persistence in the oral environment.

The resilience of oral biofilms and their associated pathologies underscore the urgency for innovative therapeutic strategies.^4^ Delivering drug and active agents to combat biofilm infections is complicated by the dynamic nature of the biological barriers within the oral cavity. Moreover, the oral cavity presents surfaces with widely varying properties. The mineralized hard surface of tooth enamel has micro-scale roughness, whereas the mucosal surface is soft and highly hydrated.^5,6^ Artificial materials such as resin are present in a large portion of the population from dental fillings, implants, or dentures.^7,8^ The ability to target these surfaces for the delivery of antimicrobial agents would greatly enhance the efficacy of preventive and therapeutic treatments.

Polymer microparticles have been widely investigated for sustained and controlled release of therapeutic agents across various medical and healthcare applications.^9–11^ By modifying their size, shape and chemical composition, it is possible to tailor the binding and drug delivery functionality of microparticles, enhancing drug loading and release kinetics to improve *in situ* bioavailability. Furthermore, the use of biodegradable and biocompatible materials in the fabrication of these microparticles has mitigated long-standing concerns related to safety and toxicity.^12^ Among several polymers that have been explored, polyethylene glycol (PEG)^13,14^ and poly(lactic-*co*-glycolic acid) (PLGA),^15,16^ both of which have FDA-approved biocompatibility, hold significant potential across a broad spectrum of clinical and therapeutic applications ranging from intravenous to pulmonary drug delivery, highlighting their versatility and safety in diverse medical settings.^16,17^ Despite these recent advances, polymer microparticles are seldom used for the delivery of therapeutic agents in the oral environment due to the aforementioned challenges; namely, the drug-carrying microparticles would be rapidly cleared from the oral cavity, dramatically reducing the bioavailability of the drug.

To address this challenge, we introduce dendritic microparticles with enhanced adhesion to oral surfaces, engineered for the delivery of antifungal agents and antibiofilm enzymes to inhibit biofilm formation. These microparticles are produced by emulsifying a blend of PLGA random copolymer (RCP) and PLGA-*b*-poly(ethylene glycol) (PLGA-*b*-PEG) block copolymer (BCP) in the oil phase of water-in-oil-in-water emulsions.^15,16^ Upon evaporation of the solvent, these emulsion droplets are converted into solid particles with a large density of long dendrites on the surface.^18^ We show that these dendritic microparticles adhere strongly onto oral surfaces such as teeth and mucosa while releasing their encapsulated antifungal agents such as a hydrophobic antifungal agent, nystatin, and a biofilm disrupting enzyme, lipase, to prevent the adhesion of *C. albicans* and their conversion to hyphae, a virulent form of the fungus. While this study focuses on oral applications, the potential of these microparticles extends to various areas, including food, personal care and pharmaceutical applications.

## Experimental

### Materials

PLGA_35k–45k_ (*M*_w_ = 35 000–45 000) and PLGA_40k_-*b*-PEG_5k_ were purchased from Nanosoft Polymers. All PLGAs were composed of lactide and glycolide at a 50 : 50 ratio. Poly(vinyl alcohol) (PVA, *M*_w_ = 13k–23k, 87–89% hydrolyzed), dichloromethane (DCM) and lipase from *Aspergillus niger* (powder (fine), ∼200 U g^−1^) were purchased from Sigma-Aldrich.

### Fabrication of drug-loaded dendritic nanoparticles

We prepared a PVA aqueous solution containing 20 mg mL^−1^ of the antifungal drug nystatin in a 2.5 wt% PVA solution. Subsequently, 100 μL of the prepared solution was combined with 1 mL of DCM containing BCP and RCP at a concentration of 10 mg mL^−1^, using vortexing to create a water-in-oil (W/O) single emulsion. Utilizing this W/O single emulsion as the dispersed phase in a 0.1 wt% PVA continuous phase aqueous solution, we obtained a W/O/W double emulsion. This double emulsion was then gently poured into a PVA aqueous solution at 60 °C with a pH higher than 8.5 to prevent dewetting of the emulsion droplets.^19^ Subsequently, the double emulsion was heated at 60 °C with magnetic stirring, inducing the evaporation of the middle layer, DCM, and resulting in the formation of drug-loaded dendritic microparticles. We characterized the synthesized dendritic microparticles using two types of scanning electron microscopes (SEMs): FEI Quanta 600 (FEI, Portland, OR, USA) and JSM-7500F (JEOL, Japan) and a Zeiss Axio Zoom.V16 fluorescence upright stereo zoom microscope (Carl Zeiss Microscopy GmbH, Jena, Germany), equipped with a 1× objective lens (numerical aperture, 0.25) was also used.

### Adhesion properties of dendritic microparticles

We fabricated individual 3D-printed acrylic substrates as model surfaces, each measuring 12.2 mm in diameter and 1.7 mm in thickness. These dimensions were identical to those of hydroxyapatite discs (Clarkson Chromatography Products, Inc., South Williamsport, PA, USA), which were used to simulate the non-biological surfaces of dentures in the oral cavity. 3D printing was conducted using a Form 3B low-force stereolithography (SLA) printer (Formlabs Inc., MA, USA), employing a biocompatible Surgical Guide resin. The 3D-printed components were cleaned in 99.9% isopropanol for 20 min and subsequently exposed to ultraviolet light (405 nm at 70 °C) for 30 min for photopolymerization (FormCure, Formlabs Inc., MA, USA). Post-printing, the slabs were polished using a muslin wheel (Kerr Dental, USA) and fine pumice (Benco, USA), followed by 5-minute bath sonication to remove any residual pumice.

Extracted human incisor teeth, procured for surgical reasons from the School of Dental Medicine, were repurposed in this study, ensuring that no patient identifiers were included. Mucosal tissue samples, exceeding 4 mm by 2 mm in size, were harvested from the palates of C57BL/6 mice. These samples were then cultivated in MEM α media containing 15% FBS, 2 mM l-glutamine, 100 μM ascorbic acid, 100 U mL^−1^ penicillin, and 100 μg mL^−1^ streptomycin, and incubated at 37 °C in a 5% CO_2_ atmosphere for 24 h.

The dendritic and spherical microparticles embedded with Nile-red underwent three centrifugation cycles at 800*g* for 10 min each, using Milli-Q water for washing. Post-centrifugation, the precipitated microparticles were resuspended in 1 mL of Milli-Q water. We then extracted 30 μL of this suspension (10 μL for mucosal tissue applications) and deposited it onto the surfaces of saliva-coated dental resin (sResin), saliva-coated hydroxyapatite discs (sHA), saliva-coated extracted human teeth (enamel surface), and oral mucosal tissue, allowing for an adhesion time of 2 min. The adhesion of these microparticles was initially visualized using a fluorescence microscope. To evaluate their adhesive strength, we subjected the particles to shear stress by immersing the samples with attached microparticles in 80 mL of Milli-Q water and agitated them at 500 rpm in a 120 mL beaker. This process lasted for 10 min for most surfaces and 1 min for mucosal tissues. Subsequently, the microparticles were re-examined under a fluorescence microscope to assess if they maintained their adhesive capabilities and remained adhered to the surface.

To quantify the coverage of the dendritic microparticles attached to the substrates, we utilized the open-source ImageJ (Fiji) software. The processing of images involved isolating the red channel (Nile red) from the stereo microscopy data. We defined a standardized, constant rectangular region of interest (ROI; 1600 × 1600 μm^2^ for sHA and sResin, 720 × 720 μm^2^ for tooth and mucosal tissue) to exclusively analyze and compare the biofilm area on the substrates. To minimize noise and mitigate false segmentation of the background and out-of-focus signals, a median filter with a radius of 2 was employed. The microparticle-bound areas within the ROIs were then classified and segmented using Otsu's automatic global thresholding algorithm,^20^ as implemented in Fiji. To evaluate the binding efficacy of the dendritic microparticles under vigorous conditions, we calculated it as the ratio A_status_/A_initial_, where “A_status_” represented the segmented area of dendritic microparticles remaining post-washing, and “A_initial_” denoted the segmented area of dendritic microparticles immediately after adhesion. This ratio served as a normalized measure of the dendritic microparticle area that remains attached.

### Biodegradability of dendritic microparticles

Nile-red embedded dendritic microparticles were synthesized and subjected to a washing process involving three rounds of centrifugation at 800*g* for 10 min each, using Milli-Q water for purification. After centrifugation, the settled microparticles were resuspended in 1 mL of Milli-Q water. A 30 μL aliquot of this suspension was then applied to a saliva-coated tooth surface (enamel) and allowed to adhere for a period of 2 min. The initial status of these microparticles was examined using fluorescence microscopy. To investigate their biodegradable characteristics, the treated tooth was immersed in 2.5 mL of Milli-Q water in a 24-well plate and maintained at room temperature. This immersion was continued for a duration of 18 days. The microparticles were then re-evaluated under a fluorescence microscope on the 9th and 18th days post-application to monitor any degradation or persistence of adhesion on tooth enamel. We also tested biodegradability in saliva following the same procedure as described above using sHA disks. The treated sHA disks were immersed in 5 mL of Milli-Q water and whole saliva at 37 °C.

### Encapsulated drug release from dendritic microparticles

In this study, we evaluate the release of nystatin from the spherical and dendritic PLGA microparticles. For nystatin, microparticles encapsulating 25 mL of 20 mg mL^−1^ nystatin were prepared and centrifuged at 800*g* for 10 min, repeated three times with Milli-Q water, to remove any unencapsulated drug, residual polyvinyl alcohol (PVA), and NaOH. Post-centrifugation, these microparticles were diluted to a 5 mL volume in a 50 mL centrifuge tube, suitable for subsequent optical analysis. The solution was incubated at 37 °C in a rocking environment. For UV-Vis spectrophotometry, a 250 μL aliquot of the incubated solution was taken periodically (every 24 h) and centrifuged (1300*g*, 10 s) to separate the supernatant from the microparticles. A 200 μL sample of the supernatant was then analyzed using a Nanodrop spectrophotometer (Nanodrop 2000 Spectrophotometer, Thermo Scientific). The amount or ratio of released nystatin was calculated based on the initial encapsulation amount and the standard curve of nystatin, with a control sample of drug-free microparticles included for background data correction.

### Biofilm inhibition using microparticles

*Candida albicans* SC5314, a well-documented opportunistic fungal pathogen, was cultivated to its mid-exponential phase (absorbance at 600 nm was 0.65) in ultrafiltered (10-kDa molecular-mass cutoff membrane; Millipore, MA, USA) tryptone-yeast extract broth (UFTYE, pH 5.5) supplemented with 1% (wt/vol) glucose at 37 °C and 5% CO_2_ as previously detailed.^21^ The cells of *C. albicans*, encompassing both yeast and hyphal forms, were collected through centrifugation (6000*g*, 10 min, 4 °C) as described previously^22^ and then suspended in 0.1 M sodium acetate (pH 4.5). To mimic dental enamel surface, hydroxyapatite discs (surface area, 2.7 ± 0.2 cm^2^; Clarkson Chromatography Products, Inc., South Williamsport, PA), coated with filter-sterilized, clarified whole saliva, were vertically arranged in a 24-well plate using a custom-built disc holder.^23^ The synthesized drug-loaded or non-drug-loaded dendritic microparticles were subjected to a washing process involving three rounds of centrifugation at 800*g* for 10 min each, using sterile phosphate-buffered saline (PBS) for purification. After centrifugation, the settled microparticles were resuspended in 1 mL of PBS. A total of 30 μL aliquot of this suspension was then topically applied to sHA for 2 min. These microparticle-treated sHA and pristine sHA discs were then inoculated with approximately 10^6^ CFUs (colony-forming units) per milliliter of *C. albicans* in UFTYE (pH 7.0) supplemented with 1% (w/v) sucrose and incubated at 37 °C in a 5% CO_2_ environment for 19 h.

To evaluate the antifungal efficacy of the dendritic microparticles, we employed a standard culturing method (quantifying viable cells *via* CFU count) alongside stereoscope-based fluorescence imaging. During the culturing process, the overall CFU count per biofilm was calculated post-biofilm treatment. In brief, the biofilm was removed from the sHA discs and homogenized *via* water bath sonication followed by probe sonication (30 s pulses at 7 W power; Branson Sonifier 150; Branson Ultrasonics, CT, USA). The resultant homogenized biofilm suspension was serially diluted and plated, and the number of viable cells was determined through CFU enumeration, as detailed previously.^24^ To analyze the fungal biofilm, *C. albicans* cells were stained using concanavalin A–tetramethyl rhodamine conjugate (Molecular Probes), and high-resolution fluorescence images were acquired using confocal laser scanning fluorescence microscope (LSM800 with Airyscan, Zeiss, Germany), equipped with a 20× water immersion objective (numerical aperture 1.0). The confocal images were then subjected to quantitative computational imaging using ImageJ (Fiji) and Otsu's thresholding algorithm to determine the biovolumes of *C. albicans* using an optimized protocol for biofilm analysis.

### Statistical analysis

Statistical analyses were performed with GraphPad Prism 8.0 (GraphPad Software, Inc., La Jolla, CA). All data are represented as mean ± SD. Comparisons of mean between multiple groups were performed using a one-way analysis of variance with *post hoc* Tukey's test, where *P* < 0.05 was considered significant and *P* > 0.05 was considered not significant. At least three independent experiments were performed unless otherwise stated.

## Results and discussion

### Fabrication of drug-loaded dendritic microparticles

**Fig. 1:**
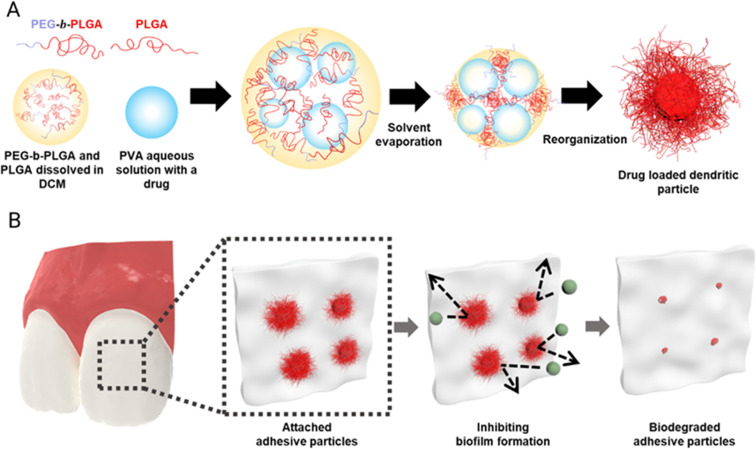
Schematic illustrations of drug-loaded dendritic microparticles. (A) The synthesis process of dendritic microparticles and (B) their attachment to dental surfaces for *in situ* drug delivery followed by biodegradation.

We fabricate dendritic microparticles by producing a water-in-oil-in-water (W/O/W) emulsion with a mixture of amphiphilic BCP and RCP dissolved in an organic solvent, and subsequently evaporating the solvent, as illustrated in Fig. 1A. The W/O/W emulsion allows for the incorporation of the antifungal agent, Nystatin, a biofilm-removing enzyme lipase, in the inner water phase. Prior studies have shown that when an oil-in-water (O/W) emulsion is prepared with an amphiphilic BCP with an appropriate hydrophilic–hydrophobic block ratio (typically 1 : 2.5–1 : 5) and a volatile organic solvent as the oil phase and the solvent is removed, the amphiphilic BCPs undergo assembly, inducing the formation of dendritic microparticles. It is believed that interfacial instability caused by the extremely low interfacial tension that develops during solvent evaporation along with the assembly of the BCP leads to the formation of these thin and long dendrites. To make biocompatible dendritic microparticles, we use a mixture of PLGA_40k_-*b*-PEG_5k_ BCP and PLGA_35k–45k_ RCP. Mixing PLGA_40k_-*b*-PEG_5k_ and PLGA_35k–45k_ provides a simple yet effective approach to tailor the resulting morphologies of microparticles. We hypothesize that dendritic microparticles would be highly adhesive to different oral surfaces, akin to the superb adhesion that is displayed by the gecko, which derives its high adhesivity from the hair-like follicles on its feet.^25^ Recent studies have demonstrated that dendritic microparticles produced using the delayed precipitation of polymer in solvent/non-solvent show superb adhesion properties.^25^ Our current study focuses on testing the ability of dendritic microparticles to release antifungal agents to prevent biofilm growth on oral surfaces, as schematically illustrated in Fig. 1B.

**Fig. 2:**
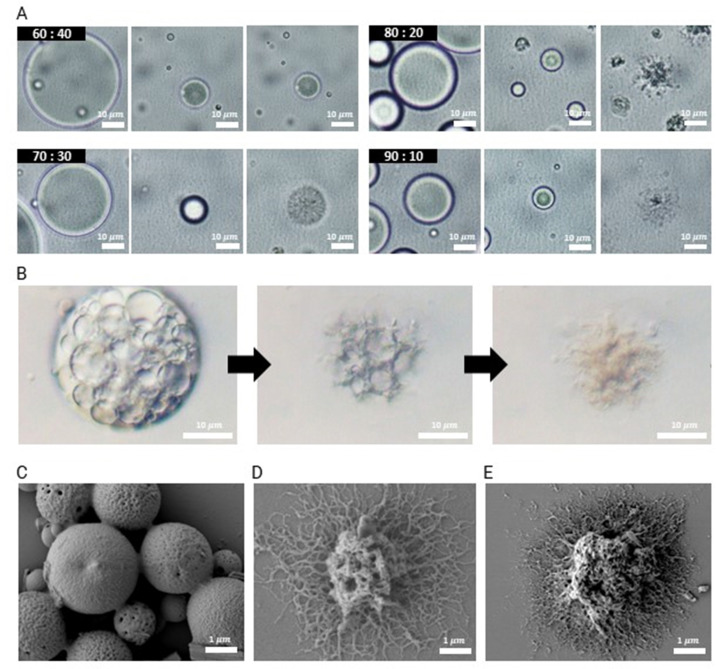
Optimizing the formation of dendritic microparticles and preparing drug-loaded dendritic microparticles. (A) Optical microscopy images depicting the extraction of solvent and dendritic particle formation through the O/W single emulsions system from the DCM emulsion droplets containing PLGA_40k_-*b*-PEG_5k_ and PLGA_35k–45k_ in the 60 : 40 to 90 : 10 ratio. The middle images of each blend show the shrunk emulsion occurring just before particle deformation due to evaporation. (B) Optical microscopy images showcasing the drug-loaded W/O/W double emulsion and providing insights into the interfacial behaviors of the double emulsion droplets containing drug at a concentration of 20 mg mL^−1^. (C)–(E) Scanning electron microscopy images were taken for the W/O/W double emulsion processed with a blend of PLGA_40k_-*b*-PEG_5k_ and PLGA_35k–45k_, both at a concentration of 10 mg mL^−1^ in DCM. The blend ratios are represented as (C) 70 : 30, (D) 80 : 20, and (E) 90 : 10, respectively.

Given the potential importance of the morphology of these microparticles in determining their adhesiveness on various oral surfaces, we study the impact of the ratio of the two polymers – PLGA_40k_-*b*-PEG_5k_ and PLGA_35k–45k_ – on the particle morphology using O/W single emulsions. O/W emulsion droplets are produced by homogenizing a mixture of PLGA_40k_-*b*-PEG_5k_ and PLGA_35k–45k_ at a total polymer concentration of 10 mg mL^−1^ dissolved in dichloromethane (DCM) in a 0.1 wt% PVA aqueous solution. The volumetric ratio of the oil to aqueous phases is 1 : 10. The removal of the solvent leads to the formation of dendrites. Dendritic structures start to emerge when the diameter of the droplets decreases by approximately 60%, as shown in Fig. 2A. To induce interfacial instability for dendritic particle formation, we find that it is essential to evaporate the solvent rapidly by applying vigorous stirring along with heating at 60 °C.

The structure of the resulting microparticles is determined by adjusting the mass ratio of PLGA_40k_-*b*-PEG_5k_ to PLGA_35k–45k_. When the mass ratio of PLGA_40k_-*b*-PEG_5k_ to PLGA_35k–45k_ is 60 : 40, microparticles formed upon solvent evaporation exhibit spherical morphology, and 70 : 30 has porous morphology with no clear dendrite formation, as shown in Fig. 2A. When the ratio of PLGA_40k_-*b*-PEG_5k_ to PLGA_35k–45k_ is increased to 80 : 20 and 90 : 10, dendritic microparticles are obtained upon the complete removal of DCM. As the total concentration of the two polymers increases from 10 mg mL^−1^ to 30 mg mL^−1^, polymer microparticles with enhanced dendrite growth are obtained, as shown in Fig. S2–S4 (ESI†).

To produce dendritic microparticles incorporating antibiofilm agents, we produce a W/O/W double emulsion, as illustrated in Fig. 1B. The inner aqueous phases are prepared using lipase dissolved in a 2.5 wt% PVA solution at a concentration of 7.5 mg mL^−1^ and nystatin dispersed in a 2.5 wt% PVA solution at a concentration of 20 mg mL^−1^. This solution is homogenized in an oil phase with PLGA_40k_-*b*-PEG_5k_ and PLGA_35k–45k_ (80 : 20 ratio, total polymer concentration = 10 mg mL^−1^) dissolved in DCM *via* vortex mixing. Thus, a stable W/O emulsion without coalescence is formed. By vortex mixing this W/O single emulsion into a 0.1 wt% PVA aqueous solution at a 1.1 : 10 volume ratio, we obtain a stable W/O/W double emulsion, as shown in Fig. S1 (ESI†). To retain the encapsulated nystatin and lipase, it is critical to control the stability of the double emulsions, which we achieve by adjusting the pH of the continuous phase to 8.5. This slightly basic condition catalyzes the hydrolysis of PLGA and the ionization of carboxylic acid end groups, enhancing the surface activity of PLGA and stabilizing the double emulsions as shown in Fig. 2B. Consistent with the morphology obtained from single emulsions, microparticles that emerge from double emulsions have dendritic structures when the ratio of PLGA_40k_-*b*-PEG_5k_ to PLGA_35k–45k_ is increased above 80 : 20, as shown in the scanning electron microscopy (SEM) images of dried microparticles in Fig. 2C–E. Furthermore, we characterized these dendritic particles, fabricated using the 80 : 20 blend ratio, by measuring their particle size and hair length (Fig. S5, ESI†). However, it is challenging to produce uniform dendritic microparticles using the current approach. To produce uniform microparticles, microfluidic emulsions can be used as templates, which would enable more detailed studies on the effect of particle size on the release and adhesion properties of these dendritic microparticles.^26^

### Adhesion properties of dendritic microparticles

To evaluate the potential of these dendritic microparticles as adhesive drug delivery vehicles in the oral cavity, we conduct adhesion tests on various surfaces found in the oral environment, including sResin, sHA, saliva-coated extracted human tooth, and oral mucosal tissue. We also synthesized equivalent-size spherical PLGA microparticles with a smooth surface as a control using the W/O/W double emulsion system. RCPs without BCPs are dissolved in DCM (PLGA_40k_-*b*-PEG_5k_ and PLGA_35k–45k_ (0 : 100 ratio), total polymer concentration = 10 mg mL^−1^), and under the same evaporating conditions, spherical microparticles are obtained (Fig. S7, ESI†). Nile red is added to the microparticles to facilitate visualization using fluorescence microscopy. Thirty microliters of this suspension (10 μL for mucosal tissue applications) at 1 wt% are deposited onto each surface and allowed to adhere to the surface for 2 minutes. The microparticles on the surfaces are subjected to shear stress by immersing them in 80 mL of water, which is stirred at 500 rpm using a magnetic stirrer in a 120 mL beaker for 10 min (1 minute for mucosal tissues).

**Fig. 3:**
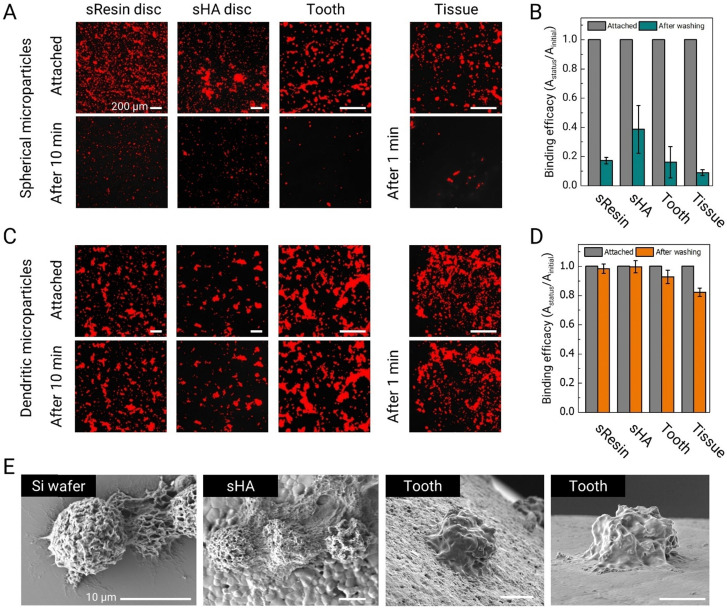
Adhesive properties and adhesion stability of dendritic microparticles to various surfaces in the oral environment. (A) Fluorescence microscopy images of spherical PLGA microparticles (∼10 μm) labelled with Nile red after adhesion to sResin, sHA, saliva-coated tooth (enamel surface) and mucosal surfaces, and after removal by washing for 10 and 1 min, scale bars are 200 μm. (B) Binding efficiency calculated by image analysis of fluorescence microscopy images before and after washing of PLGA microparticle. (C) Fluorescence microscopy images of dendritic microparticles to compare their adhesive properties in an equivalent environment to spherical microparticles, scale bars are 200 μm. (D) Binding efficiency calculated by image analysis of fluorescence microscopy images before and after washing of dendritic microparticles. (E) SEM images of dendritic microparticles adhering to Si-wafer and various dental surfaces, scale bars are 10 μm. Data are mean ± standard deviation.

Fluorescence microscopy images reveal that a substantial fraction of spherical PLGA microparticles becomes easily dislodged under the influence of the shear flow generated by the magnetic stirrer (Fig. 3A). The shear stress in this test is approximately 0.015 Pa, which falls within the range of shear stress generated by salivary flow within the mouth.^27^ Quantitative analysis of these images further supports our observations. More than 80% of smooth spherical microparticles detached from the sResin and tooth surfaces. On sHA, a larger fraction of microparticles are retained, likely owing to its higher surface roughness; nevertheless, more than half (∼60%) are removed. On the mucosal surface, which is a soft surface, over 90% of the microparticles detach, showing very poor adhesion and retention (Fig. 3B).

We subsequently assess the adhesion performance of dendritic microparticles made with a blend of PLGA_40k_-*b*-PEG_5k_ and PLGA_35k–45k_ (80 : 20 ratio) under equivalent test conditions. The dendritic microparticles maintain stable adhesion to all tested oral surfaces, as seen in Fig. 3C. Specifically, over 99% of dendritic microparticles remain adhered on sHA surfaces, while more than 98% remain adhered to sResin, and 92% remain on tooth surfaces (Fig. 3D). Remarkably, over 82% of the microparticles exhibit robust adhesion to mucosal surfaces. The adhesive properties of these dendritic microparticles can be attributed, at least in part, to substantial van der Waals forces facilitated by the widespread contact between their tentacle-like dendrites and the target surfaces. SEM images provide a detailed visualization of the adhesion of dendritic microparticles to various surfaces, illustrating the conformal adhesion of dendrites to these surfaces (Fig. 3E).

### Encapsulated drug release and biodegradability of dendritic microparticles

**Fig. 4:**
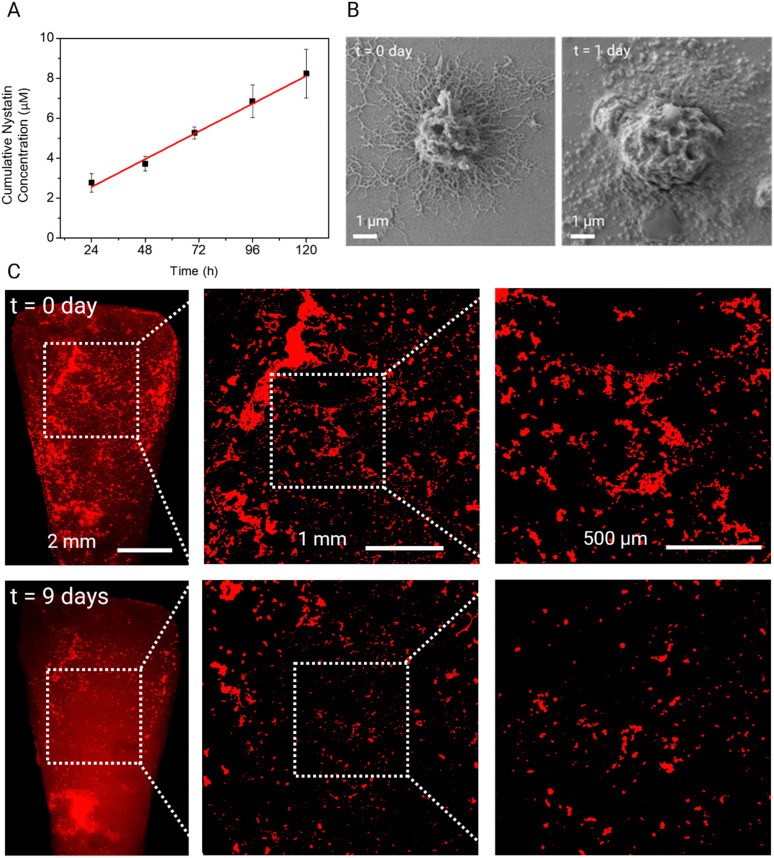
Dendritic microparticle degradation and drug release. (A) Cumulative concentration release curve of nystatin from the dendritic microparticles at 37 °C under stirring. The fit of the mathematical model to the experimental data is presented by a red solid line. Data are mean ± standard deviation. (B) SEM images showing the polymer degradation of dendritic microparticles after 24 h in water. (C) Fluorescence images of dendritic microparticles on a saliva-coated human tooth for 0 and 9 days.

To investigate the release kinetics of these dendritic microparticles, the nystatin-loaded dendritic microparticles are placed in deionized water at 37 °C under gentle stirring. To quantify the nystatin concentration released from the dendritic microparticles, time-dependent absorbance spectra are measured for 5 days (Fig. S6, ESI†). As shown in Fig. 4A, nystatin is released gradually over time with a linear profile over 5 days. Such a linear profile has been reported previously for release from PLGA microparticles. Interestingly, extrapolation to 0 h gave a non-zero intercept, indicating that there was likely a burst release of the drug in the first few hours, followed by slow, sustained release, which is likely advantageous for dental applications. SEM observation of dendritic microparticles shows that the hydrolysis of PLGA affects the morphology of dendrites. Many dendrites lost their definitive features after 24 h; nevertheless, microparticles remained well bound on the surface (Fig. 4B). The PLGA spherical microparticles show a similar nystatin release profile under the same experimental conditions (Fig. S7 and S8, ESI†), indicating that the morphology of the particles does not significantly affect the drug release behavior of these PLGA microparticles. We also assessed the time-dependent biodegradation of dendritic microparticles on tooth surfaces. Fluorescence microscopy data show degradation of dendritic microparticles adhered to tooth surfaces, resulting in a reduction in the quantity of attached microparticles, as shown in Fig. 4C. Notably, a significant portion of these adherent microparticles disappeared from the tooth surface by day 18, as depicted in Fig. S9 (ESI†). The biodegradation behavior of dendritic microparticles on sHA disks is also tested in ultrapure water and whole saliva at 37 °C (Fig. S10, ESI†). The dendritic microparticles show a similar time-dependent biodegradation behavior in both conditions.

### Biofilm inhibition of drug-loaded dendritic microparticles

**Fig. 5:**
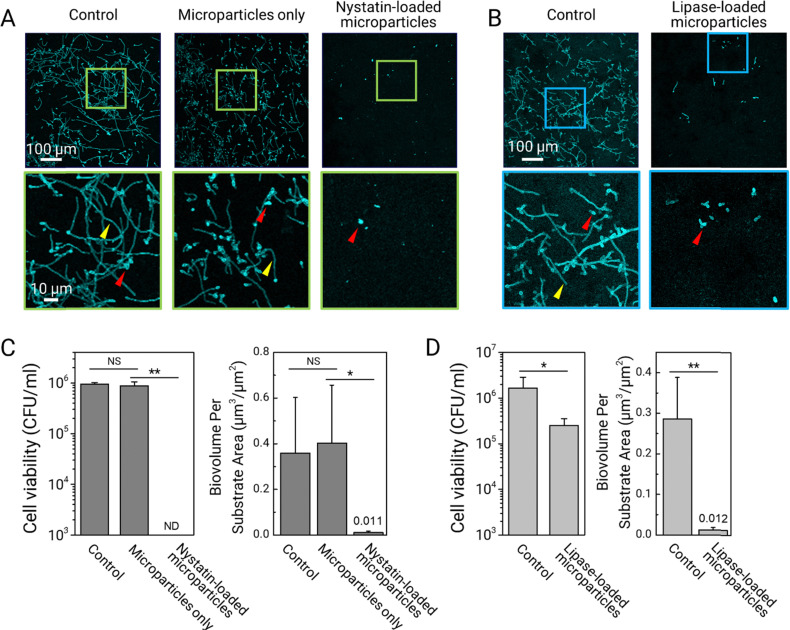
Antibiofilm effects of nystatin- or lipase-loaded dendritic microparticles against fungal biofilms. (A) and (B) Confocal images demonstrate the antifungal efficacy of nystatin- or lipase-loaded dendritic microparticles. *C. albicans* cells (yeasts (red arrowheads) or hyphae (yellow arrowheads)) are depicted in cyan. Magnified (close-up) view of each small box positioned in the bottom panel. (C) and (D) Cell viability counts show killing of targeted fungal biofilms *via* attached dendritic microparticles with loaded nystatin or lipase (*n* = 4). Quantitative computational analysis of the confocal fungal biofilm images treated with nystatin- or lipase-loaded dendritic microparticles (*n* = 3). ND means no detectable. Data are mean ± standard deviation, *P < 0.05; **P < 0.01; NS means not significant *P* > 0.5; one-way analysis of variance followed by Tukey's multiple-comparison test.

We assess the biofilm prevention activity of drug-encapsulated dendritic microparticles using a *C. albicans* biofilm model (see the Experimental section). Before inoculation with *C. albicans*, sHA discs are topically treated with the prepared drug-loaded and non-drug-loaded dendritic microparticle suspensions. To serve as vehicle control, dendritic microparticles without drug payloads are used in the study. Then, each of the treated and non-treated sHA discs is inoculated with actively growing *C. albicans*. For biofilm formation, the surfaces are incubated at 37 °C in UFTYE supplemented with 1% (w/v) sucrose and 5% CO_2_ for 19 h. The data reveal that treatment with dendritic microparticles loaded with nystatin significantly inhibits *C. albicans* (in cyan) accumulation and hyphal formation (Fig. 5A). Fungal cells are in yeast form (Fig. 5A, bottom row, red arrowheads) with no detectable hyphae. Moreover, the cell viability data indicate the complete killing of the fungal cells following treatment (Fig. 5C, left). Further analysis confirms the inhibitory effects exerted by the nystatin-loaded dendritic microparticles, showing negligible biofilm biomass (biovolume) compared to the untreated control or vehicle control (Fig. 5C, right).

To investigate whether dendritic microparticles can deliver therapeutic enzymes, we perform the bioassays using lipase-loaded dendritic microparticles. Dendritic microparticles loaded with lipase are effective in reducing fungal accumulation and limiting hyphal development, as shown in Fig. 5B. However, the lipase-loaded microparticles are unable to completely kill *C. albicans* (Fig. 5D), indicating that the fungal cells (primarily yeast forms) attached to the treated surface are mostly viable. This observation is consistent with the lack of fungicidal activity of lipase while blocking hyphae formation.^28^ Nevertheless, these results demonstrate a significant ability to reduce biofilm biomass accumulation due to effective inhibition of hyphal development (Fig. 5D). Altogether, our results show the efficacy of dendritic microparticles in the sustained delivery of drugs or biologics to various oral surfaces, resulting in efficient biofilm control. Expanding upon these findings, our future work will involve conducting comprehensive biocompatibility tests on these microparticles, which is a critical step to ensure their safety for clinical use.

## Conclusions

In summary, our study presents a novel approach to address the persistent challenge of biofilm-associated infections in the oral cavity through the development of adhesive microparticles. These microparticles, characterized by their unique dendritic surface structures, demonstrate exceptional adhesion to various oral surfaces, including dental resin, hydroxyapatite, tooth enamel, and mucosal tissues. This adhesion capability is a critical factor in their effectiveness, as it ensures the sustained presence and gradual release of therapeutic agents in the oral environment, which is a key requirement given the dynamic and complex nature of this biological setting. The incorporation of antifungal agents such as nystatin and biofilm-disrupting enzymes like lipase into these microparticles demonstrates significant promise in inhibiting the growth and virulence of *C. albicans* biofilms. The sustained release profile of these agents from the dendritic microparticles, coupled with their biodegradable nature, offers the dual advantages of prolonged efficacy and safety. Our findings indicate that these microparticles can effectively prevent the formation of fungal biofilms and limit the transition of *C. albicans* into its more virulent hyphal form. We believe that the versatility of these dendritic microparticles extends beyond oral healthcare applications. Their potential utility in various fields, such as food preservation, personal care products, and broader pharmaceutical applications, underscores their significance. The ability to tailor the microparticles’ composition and morphology for specific applications, as well as load synthetic drugs or biologics, further enhances their appeal as a customizable solution to a range of microbial challenges.

## Author contributions

The manuscript was written through contributions from all authors. All authors have given approval to the final version of the manuscript.

## Conflicts of interest

The authors declare no competing financial interest.

## Supplementary Material

TB-012-D4TB00134F-s001
