# Supplementary material for: Drug-loaded adhesive microparticles for biofilm prevention on oral surfaces
Source: J Mater Chem B. 2024 Apr 29;12(20):4935–44. doi: 10.1039/d4tb00134f (PMC11111112; doi:10.1039/d4tb00134f)
Supplement: TB-012-D4TB00134F-s001 [file TB-012-D4TB00134F-s001.pdf]

Electronic Supplementary Material (ESI) for Journal of Materials Chemistry B.
This journal is © The Royal Society of Chemistry 2024

Supporting Information

Drug-loaded Adhesive Microparticles for Biofilm Prevention on Oral Surfaces

Min Jun Oh, † *Jae-Hyun Kim,* † *Jaekyoung Kim,*‡ *Sunghee Lee,*‡ *Hyun Koo,* * *Daeyeon Lee**

†These authors contributed equally to this work.

‡These authors contributed equally to this work.

Min Jun Oh, Jae-Hyun Kim, Jaekyoung Kim, Sunghee Lee, Daeyeon Lee

Department of Chemical and Biomolecular Engineering, University of Pennsylvania

Philadelphia, Pennsylvania 19104, United States

Min Jun Oh, Hyun Koo

Department of Orthodontics, School of Dental Medicine, University of Pennsylvania, Philadelphia, Pennsylvania 19104, United States

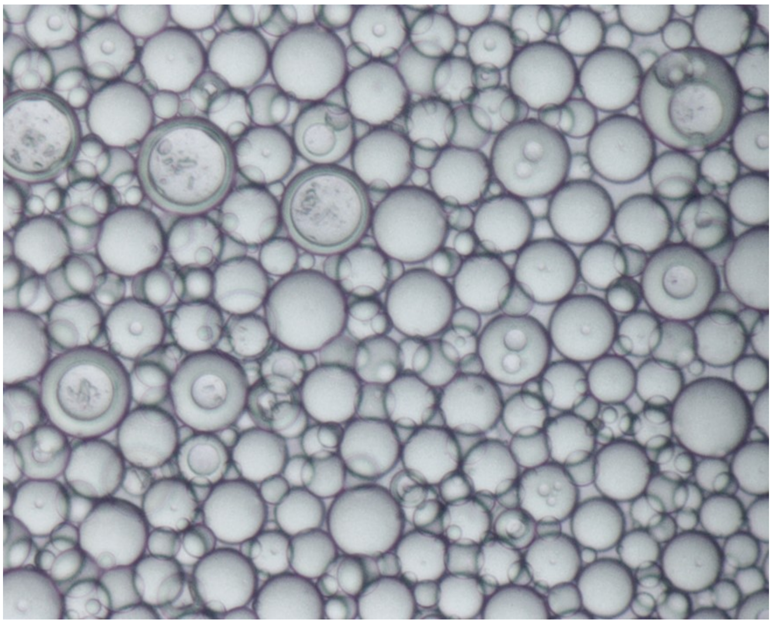

Figure S1. A microscopic image of the W/O/W emulsion with nystatin dispersed in the inner phase.

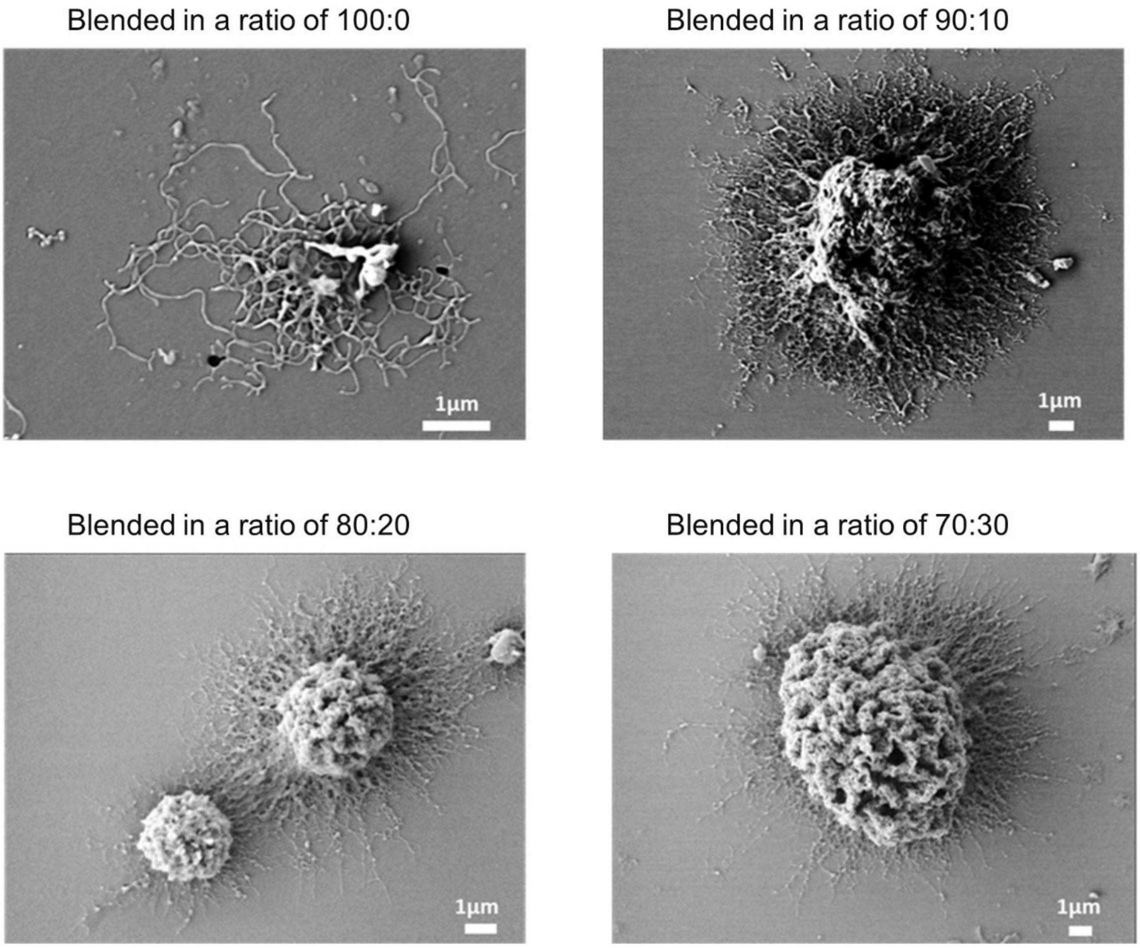

Figure S2. Fabrication of $PLGA_{40k}$-*b*-PEG_{5k} block copolymer microparticle with 10 mg/mL polymer in DCM varied by $PLGA_{35k\text{-}45k}$ blending ratio. pH at 8.5, PVA 0.1 wt% aqueous solution used as continuous phase.

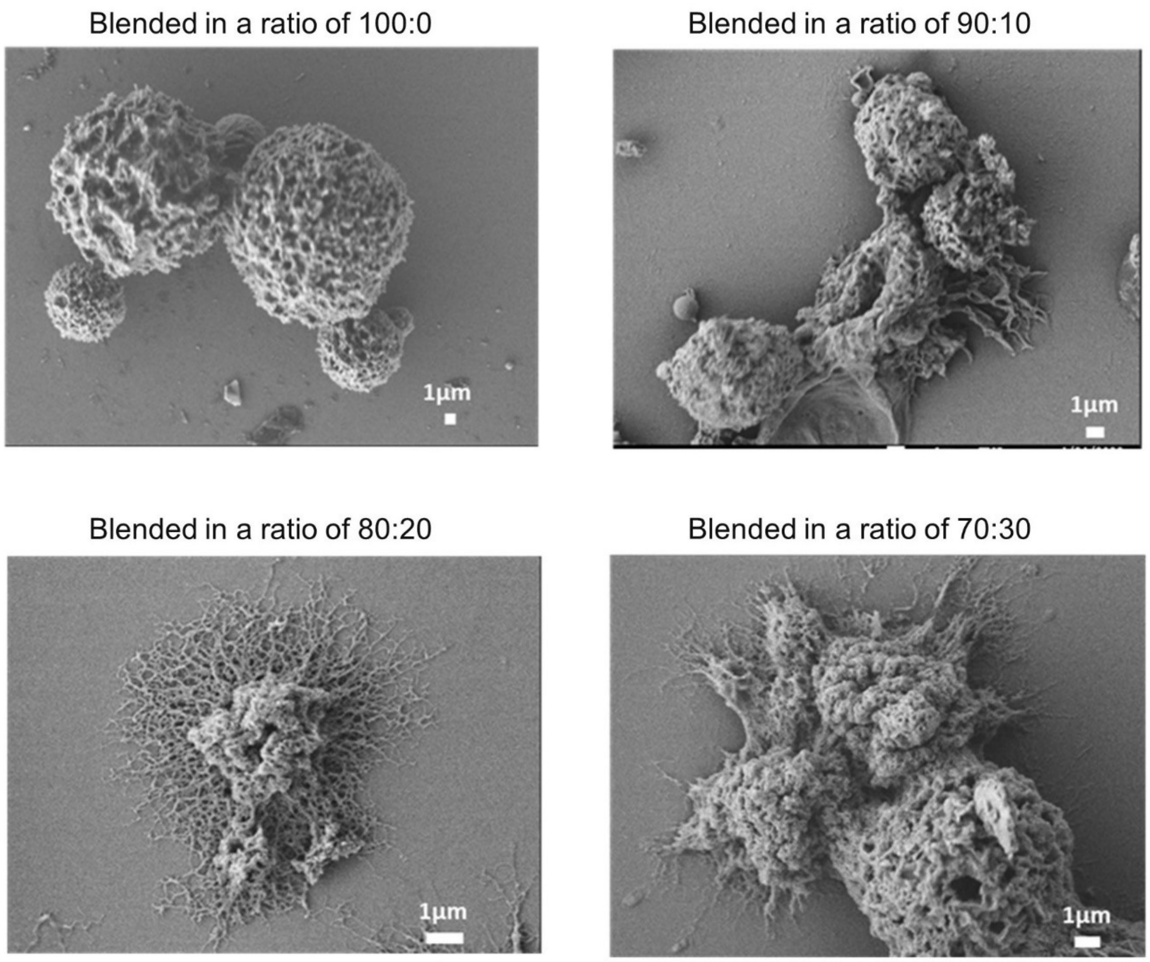

Figure S3. Fabrication of $PLGA_{40k}$-*b*-PEG_{5k} block copolymer microparticle with 30 mg/mL polymer in DCM varied by $PLGA_{35k\text{-}45k}$ blending ratio. pH at 8.5, PVA 0.1 wt% aqueous solution used as continuous phase.

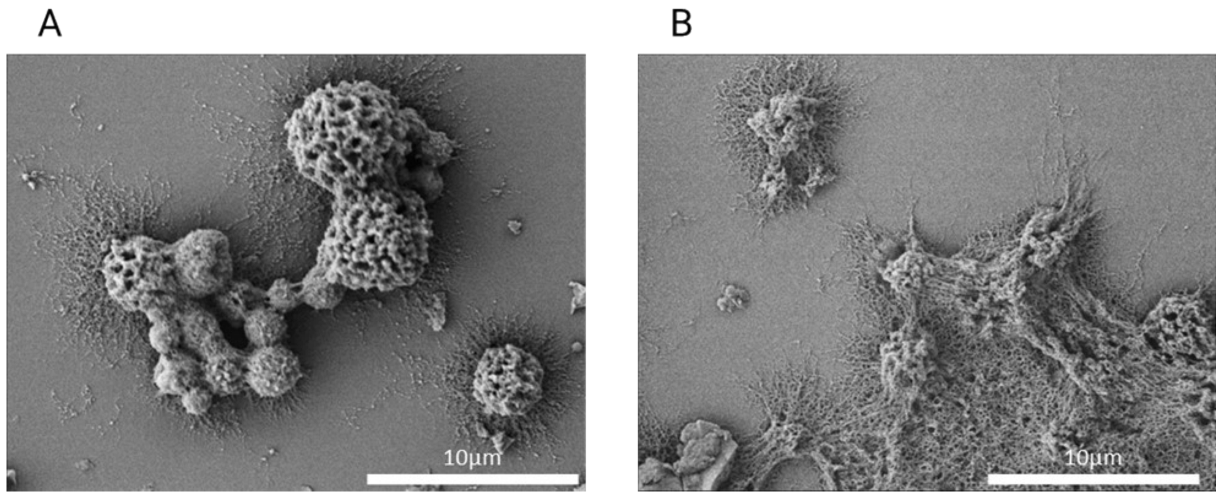

Figure S4. SEM images of $PLGA_{40k}$-*b*-PEG_{5k} and $PLGA_{35k\text{-}45k}$ blended at an 80:20 ratio, showcasing dendritic microparticles varied by the weight percentage of block copolymer, with concentrations of (A) 10 mg/mL and (B) 30 mg/mL polymer in DCM. As the polymer concentration increases from (A) to (B), the diameter of the wire branches increases and becomes more branched, which induces more physical interaction during the sample preparation.

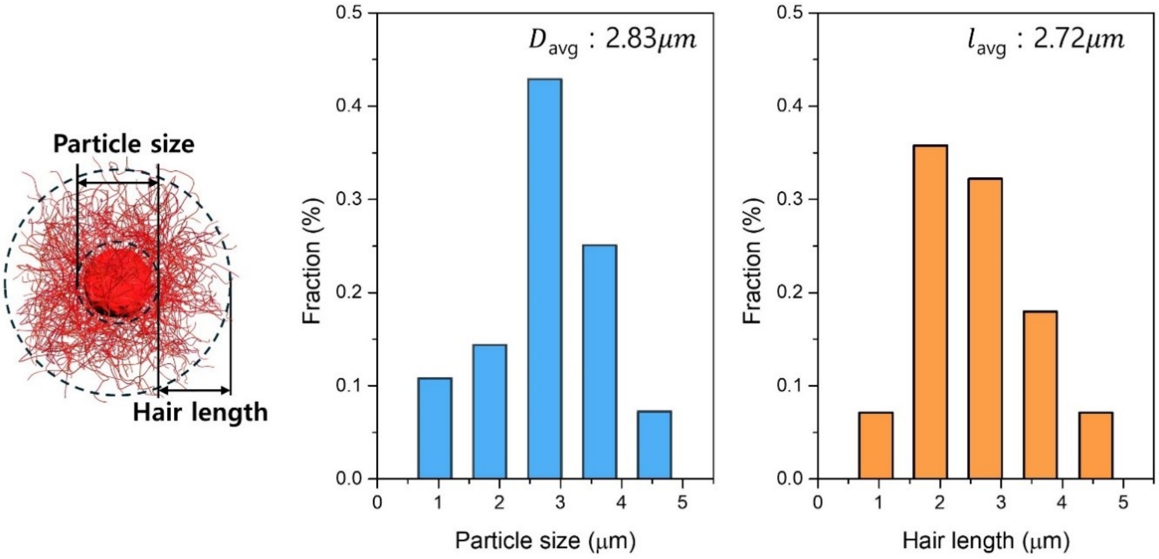

Figure S5. Histograms displaying the particle size (D) and the dendrite length (l) of drug-loaded dendritic nanoparticles, which are fabricated using a blend of $PLGA_{40k}$-b-PEG_{5k} and $PLGA_{35k-45k}$ at an 80:20 ratio, with a polymer concentration of 10 mg/mL in DCM.

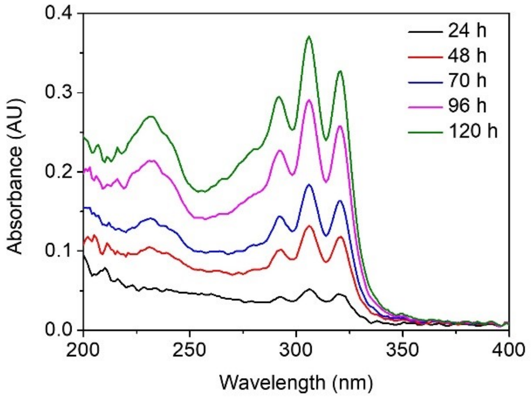

Figure S6. Time-dependent absorbance spectra of nystatin from the dendritic microparticles.

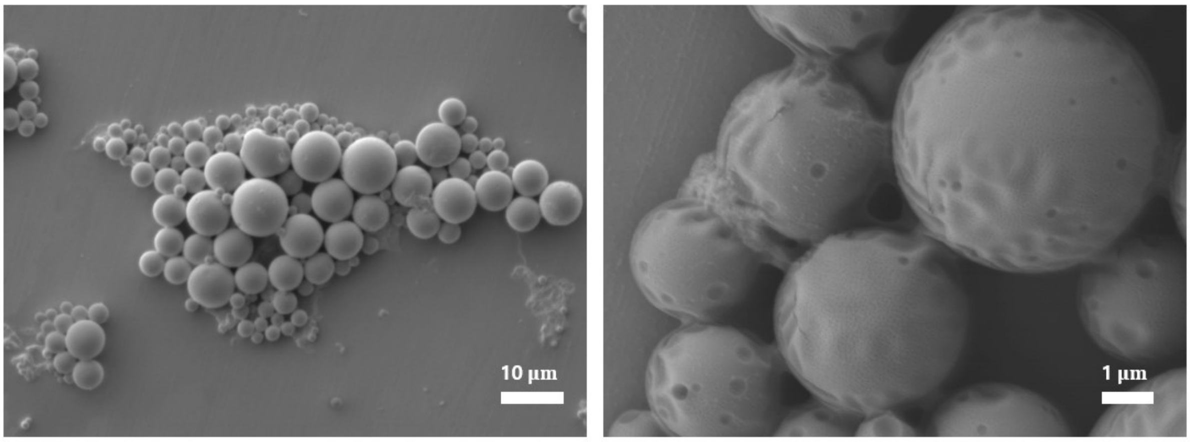

Figure S7. SEM images of spherical PLGA microparticles fabricated using the 80:20 blend ratio with 10 mg/mL polymer in DCM.

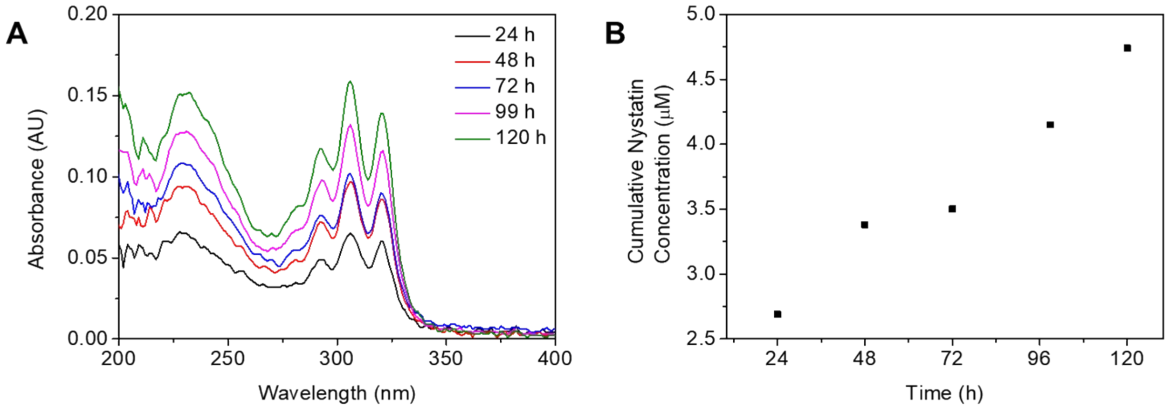

Figure S8. (A) Time-dependent absorbance spectra of nystatin from the PLGA spherical microparticles. (B) Cumulative concentration release curve of nystatin from PLGA spherical particles at 37°C under stirring.

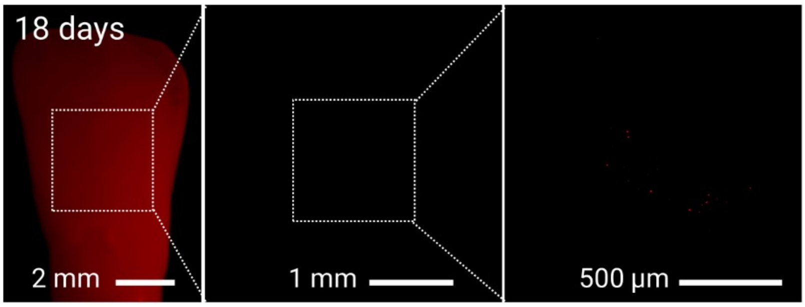

Figure S9. Fluorescence images of dendritic microparticles on a saliva-coated human tooth for 18 days.

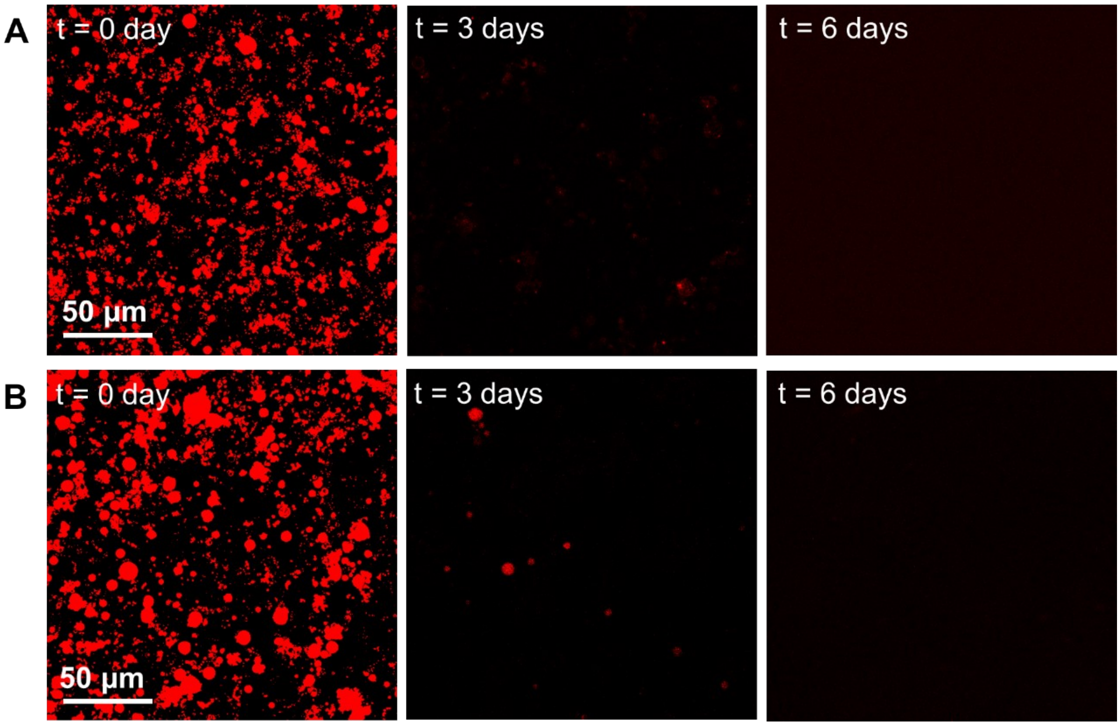

Figure S10. (A-B) Fluorescence images of dendritic microparticles on a sHA disk under ultrapure water (A) and whole saliva (B) after 0, 3 and 6 days at 37 °C. All fluorescence images share the same scale bar.
